# DNA-Detection Based Diagnostics for *Taenia solium* Cysticercosis in Porcine

**DOI:** 10.1155/2020/5706981

**Published:** 2020-01-07

**Authors:** Maxwell W. Waema, Gerald Misinzo, John M. Kagira, Eric L. Agola, Helena A. Ngowi

**Affiliations:** ^1^Southern African Centre for Infectious Disease Surveillance (SACIDS), Sokoine University of Agriculture, P.O Box 3297, Chuo Kikuu, Morogoro, Tanzania; ^2^Department of Animal Health and Production, Jomo Kenyatta University of Agriculture and Technology, P.O Box 62000-00200, Nairobi, Kenya; ^3^Centre of Biotechnology Research and Development, Kenya Medical Research Institute, P.O Box 3297, Nairobi, Kenya; ^4^Department of Veterinary Medicine and Public Health, Sokoine University of Agriculture, P.O Box 3021, Chuo Kikuu, Morogoro, Tanzania

## Abstract

Porcine cysticercosis is a neglected and underestimated disease caused by metacestode stage of the tapeworm, *Taenia solium* (*T. solium*). Pigs are the intermediate hosts of *T. solium *while human are the only known definitive host. The disease has an economic consequence because the affected farmers lose 50−100 percent of the value of pigs if they are infected. Lack of affordable, easy to use, sensitive, and specific molecular diagnostic tools for detection of infections at the farm level hinders the control of porcine cysticercosis in endemic areas. A number of DNA based diagnostic assays for the detection of *T. solium *infections in pigs have been developed and evaluated but none is applicable at low-resource areas where this disease is an endemic. This review focuses mainly on DNA based diagnostic methods, their sensitivity, specificity, and utilization at low-resource areas. We summarized data from 65 studies on the current DNA-detection based diagnostic techniques for *T. solium* cysticercosis in porcine, published in English between the years 2000–2018, identified through PubMed search engine. Of the different polymerase chain reaction (PCR) assays developed for identification of *T. solium,* the most sensitive (97−100%) and specific (100%) one is nested PCR. One study utilized loop-mediated isothermal amplification (LAMP) as a diagnostic tool for the detection of *T. solium* infections though its field use was never determined. Recombinase polymerase amplification (RPA) has been evaluated as a diagnostic tool for a variety of diseases, but has never been exploited for the diagnosis of cysticercosis/taeniasis. In conclusion, several molecular methods have been developed and evaluated in lab settings. However, there is need to validate these methods as a diagnostic tool to diagnose porcine cysticercosis in low-resource areas.

## 1. Introduction

Porcine cysticercosis is caused by the larval stage of a zoonotic tapeworm *Taenia solium* and infections involve both human and pigs. The disease remains a major public health concern in many low-income countries where pigs are raised as a food source and are kept under free-range conditions [1]. Porcine cysticercosis has been reported in many Sub-Saharan countries with prevalence proportions as high as 64% [2] in Eastern Cape Province of South Africa [3], 23.3% in the Eastern, Southern, and Western provinces of Zambia [4] and 38.4% in Congo [5]. In East African countries, the prevalence of cysticercosis among pigs in a number of areas has been reported as being approximately 20%. Studies [1, 6] reported prevalence range of 4.5−6.7% in western Kenya. In Tanzania, *T. solium* is considered widespread in the northern and southern regions based on porcine cysticercosis surveys [7–9].

Porcine cysticercosis has an economic implication because any pig carcass found to be infested with cysticerci during meat inspection is judged to be unfit for human consumption, and in some countries the whole carcass is condemned thus loss of meat leading to food insecurity [5]. Lack of affordable, easy to use, sensitive, and specific molecular diagnostic tool for detection of infections at farm level hinders the control of porcine cysticercosis in endemic areas. Effective detection of porcine cysticercosis is vital as consumption of infected pork is a direct cause of taeniosis in humans [10].

Because of the low sensitivity and specificity of the conventional diagnosis based on lingual and serological examination, molecular tools are more reliable for differential diagnosis of porcine cysticercosis. Several molecular diagnostic tools for detection of porcine cysticercosis have been developed elsewhere [11–14]; a good example is PCR based techniques that have been developed and adopted for molecular identification of porcine cysticercosis and for screening and authentication of meat [11]. Although PCR provides sensitive and reliable diagnostic results, it is laboratory based and expensive thus unsuitable for most low-income countries and inapplicable in the low resource areas where porcine cysticercosis infections are endemic. In the past few years, several isothermal nucleic acid amplification techniques that do not require the thermocycling process have been established. Loop-mediated isothermal amplification (LAMP) [15] has proven to be a much more rapid and sensitive molecular diagnostic technique when compared to PCR [16]. The method amplifies DNA with high specificity, sensitivity, and rapidity under isothermal conditions of 60–65°C.

The Recombinase Polymerase Amplification (RPA) technology first described [17] is now being evaluated as a diagnostic tool for a variety of pathogenic microorganisms including protozoal infections [18–22], and other infectious diseases. RPA assays have benefits over PCR and LAMP because they are rapid, do not require DNA purification, require less expensive equipment and can be completed directly in the field with portable equipment making RPA an ideal assay for use in point-of-care diagnostic applications [23]. This review summarizes current DNA-detection based diagnostics for *T. solium* cysticercosis in porcine.

## 2. Methodology

PubMed online database was used to search for full length original scientific articles published in English in the period 2000–2018. The following keywords were used as search terms: Porcine cysticercosis; Isothermal amplification, *Taenia solium*, and Detection of DNA. Articles not reporting original research data or written in languages other than English were excluded. Articles containing DNA techniques for the detection of porcine cysticercosis in the abstract were selected and their full content was reviewed. We also used the reference list of the relevant articles to retrieve original studies related to our work.****From the selected articles the following information was obtained; detail of publication (author), title, source, year, volume, objective of the article, diagnostic techniques, result, and conclusion.

## 3. Results and Discussion

A total of 84 full-length journal articles were retrieved and reviewed and 65 of them were selected for the report because they described subjects of interest.

### 3.1. Polymerase Chain Reaction

PCR assays have been used in the differential identification of *Taenia* species[24, 25]. Three different PCR assays (Table 1) developed for the identification of *T. solium *[14, 26, 27]were optimized [28]. Comparison of the effectiveness of these PCRs revealed that the most sensitive (97%) and specific (100%) one is nested PCR. Yamasaki et al. [14] and Mayta et al. [26] used multiplex PCR to diagnose cysticercosis with their results showing the sensitivity of multiplex PCR being lower compared with that of the copro-antigen detection test. PCR tests (conventional) have been used in the identification of uncertain *T. solium* cystic lesions in pork meat [29], validation of macroscopic diagnosis of ambiguous lesions [30, 31], and confirmation of results obtained during inspection of meat from slaughtered pigs [27].

Several gene targets have been amplified in PCR technology (Table 2). In identification of *T. solium* cysticerci from the infected and suspected pig carcasses, Kakoty et al. [29] amplified large subunit rRNA gene (TBR). Dalmasso et al. [34] extracted DNA from degenerated and calcified cysts and found positive amplification for the TRB gene. Sreedevi et al. [27] confirmed *T. solium* cysticercosis in pigs' carcasses by targeting the cytochrome c oxidase subunit 1 (Cox1) and the TBR gene with TBR primers detecting *T. solium *cysticercusDNA at a lower concentration than with Cox1 primers. In contrast, TBR primers detected *T. solium* cyst DNA at a lower concentration (10 pg) than that of the internal transcribed spacer 1 gene primer [32].

Although PCR technology provides sensitive (97%) [26] and reliable diagnostic results [14, 27, 29] it is unsuitable for most developing country settings and inapplicable in the low resource areas where porcine cysticercosis infections are endemic because it requires sophisticated equipment, such as a thermal cycler and has a long detection period of two hours [14].

### 3.2. Loop-Mediated Isothermal Amplification

Loop-mediated isothermal amplification (LAMP) is a DNA based isothermal amplification technique first described by Notomi et al. [15] and has a wide application as a diagnostic tool for variety of helminthic and protozoan infections for both humans and animals [36–41]. As shown in (Table 3), only few studies have utilized LAMP as a diagnostic tool for the detection of *T. solium* infections [12, 13, 42]. The LAMP method targeted the mitochondrial cytochrome c oxidase subunit 1 (cox1) which exists in a cell in large amounts. Nkouawa et al. [12] developed a multiplex LAMP assay in combination with a dot enzyme-linked immunosorbent assay as a fast field-based test for the differentiation of human *Taenia* species. The LAMP assay was found to be more sensitive than the multiplex PCR technique for the differential detection of *Taenia* species in stool and cysticercus samples [43]. Although LAMP has been used in the detection of *T. solium* infections, designing of appropriate primers is more complicated compared to that of PCR and RPA [42].

LAMP uses Bst DNA polymerase for strand displacement DNA synthesis along with 4 sets of primers [15]. The Bst DNA polymerase enzyme is tolerant to inhibitors present in biological matrices [38, 44] making it a gold standard method to detect pathogens in fecal, urine, and blood samples [45–47]. The LAMP assay is more sensitive (86%) and specific (100%) than the multiplex PCR technique for the differential detection of *Taenia* species in stool and cysticercus samples [43].

Since its development in the year 2000, LAMP has under gone a number of modifications making it more simple and easy to use at point-of-care [36] following the ASSURED (Affordable, Sensitive, Specific, User friendly, Rapid, and Robust, Equipment free, Deliverable to end users) [48] guidelines of the World Health Organization. The technique eliminates the requirement of sophisticated equipment like thermocyclers as it is carried out under isothermal conditions using a simple incubator, such as a water bath [21], block heater or thermos bottles [36]. LAMP assay produces magnesium pyrophosphate visible as white precipitation [49, 50], making it possible to distinguish a positive LAMP reaction from a negative LAMP reaction by visual endpoint judgment of the turbidity of these precipitate in the tube [43]. The amplification product can also be visualized by a simple color change detection system based on staining the end products with SYBR Green dye visible with the naked eye, eliminating the need of the gel electrophoresis which cannot be performed in the field [37, 51]. Although LAMP had been used in the detection of *T. solium* infections, designing of appropriate primers is more complicated compared to that of PCR and RPA. Also, it requires higher incubation temperatures ranging from 55°C to 65°C for 60–75 minutes [42].

### 3.3. Lyophilized LAMP Format

The requirement for point-of-care diagnostic test in resource limited settings requires the ability for long shelf life without any means of a cold chain [21] and as a result lyophilized LAMP formats have been developed [52, 53]. This method eliminates the need to open the reaction tubes as it requires only sample addition, reducing the possibility of contamination [45, 54]. Hayashida et al. [37] developed a direct dry LAMP format for human African trypanosomiasis ideal for bedside or sampling site operation. The reagents maintain the same reactivity as freshly prepared reagents and remain stable for up to 3 months when stored at 4°C [52]. A study by Chander et al. [53] reported that dry LAMP formulation for bacteria and viruses remain stable at room temperature for at least 6 months with no apparent loss in activity and the sensitivity remains at about 12 copies which was similar to the freshly prepared reagents for at least 3 months [53]. Lyophilized LAMP reagents can be transported by normal post mailing without dry-ice protection and stored in places where even the refrigerators are not available making it an ideal diagnostic test for a resource-limited setting where porcine cysticercosis is endemic and cold storage of reaction components might not be available [36]. Currently there is no dry LAMP format for the detection of *Taenia solium* infections in both humans and animals.

### 3.4. Recombinase Polymerase Amplification

Recombinase polymerase amplification (RPA) is one of the newer isothermal amplification strategies that can detect DNA/RNA without requiring any laboratory equipment [17] and can be performed at constant low temperature [20, 55]. In RPA, primer annealing and elongation is enzyme mediated and not temperature driven, no melting temperature requirements for primers and probes [55]. Amplification is achieved within very short time (typically <20 minutes) [55] using combination of three enzymes with each performing distinctive function during the amplification process [57]. The three enzymes are: recombinase, single strand binding protein (SSB), and strand displacing DNA polymerase [17]. These enzymes are supplied in stable dry format which allows transportation and storage without refrigeration [17]. In addition, RPA results can be visualized with naked eyes using simple lateral flow devices [17, 22].

Similar to LAMP, RPA amplification can be achieved without the need for thermocyclers and has a wide temperature range (25–42°C) [17, 22, 56], there by needing less power because they do not require initial denaturation at 95°C and cycling steps [56]. Study by Crannell et al. showed that the human body heat which is readily available and inexpensive can be harnessed for lateral flow-RPA isothermal amplification [58]. Amplification product could be achieved using nonfluorescence formats such as lateral flow assays or lateral flow strips [18–20, 55, 59]. RPA is tolerant to sample impurities and simple lysis methods such as boiling may adequately prepare samples for amplification [60]. It is also tolerant to some common PCR inhibitors such as ethanol, heparin, and hemoglobin [22]. However, RPA is inhibited by anticoagulant in case whole blood is used as a sample [22]. RPA demonstrated to operate with nucleic acid extracted from different samples matrices such as plasma [60], serum [22], stool [31, 33], urine [61], and plant tissue [62, 63].

Although RPA has been evaluated as a diagnostic tool for a variety of protozoan diseases, some helminthic infections [64, 65] and other infectious diseases [61, 66], has not been exploited for the diagnosis of cysticercosis/taeniasis (Table 4). Since *Taenia solium* infections is endemic in resource limited settings in low-income countries, development of RPA as an appropriate diagnostic test will offer support to disease prevention, treatment, and evaluation of control programs.

The comparison between the most common isothermal amplification methods and established PCR is given in Table 5.

### 3.5. Limitations and Suggestions of RPA

Due to high sensitivity (98−100%) of the RPA assay, the amplified reaction has the possibility of cross-contamination, thus the working areas must be sterilized by UV light and sterile gloves, tubes, and pipettes used throughout. The lyophilized RPA reagents need to be mixed with primers by pipetting and transferring solutions to open tubes and addition of the samples posing a serious contamination risk especially when dealing with a huge number of samples. This can be solved by use of a semiintegrated RPA method free of pipetting [71]. False positive results may be seen due to nonspecific binding of recombinase enzyme [57]. This nonspecific binding can be solved by getting the reaction enzymes separately and recombinase is mixed first with the primers before addition of the template and the other two enzymes [57]. Study by Wu et al. has shown that LF strips tend to show false positives when left in running buffer for long time [55]. This can be eliminated by diluting the amplification product to a ratio of 1 : 100 in the running buffer [20].

## 4. Conclusion

Development of point-of-care diagnostic tests is one of the most important priorities for cysticercosis management today. Molecular methods have not yet proven useful to diagnose porcine cysticercosis in resource limited settings and more research is essential to improve the real capacity. RPA has been evaluated as a diagnostic tool for a variety of protozoan, some helminthic infections, and other infectious disease but it has not been exploited for the diagnosis of *T. solium* infection both in humans and pigs. RPA being sensitive, specific, rapid, and easy to use DNA based diagnostic test will serve to facilitate rapid diagnosis and treatment of porcine cysticercosis and also evaluate control programs in endemic areas.

## Figures and Tables

**Table 1:** PCR based techniques for *Taenia solium* identification.

Method	Sample	Target gene	References
PCR	Cysticerci	TBR gene	[29]
	Cysticerci	COX1	[27]
	Cysticerci	COX1, internal transcribed spacer 1	[32]
Nested PCR	Proglottids	Tsol31	[10, 26]
	Serum	COX1	[14, 33]
Multiplex PCR	cysticerci, proglottids	COX1	[14]

**Table 2:** Oligonucleotide primers used to detect *Taenia solium* by polymerase chain reaction.

Target gene	Primer name	Primer sequence	References
Large subunit rRNA gene	TBR-3	5′-GGCTTGTTTGAATGGTTTGACG-3′	[29, 24]
	TBR-6	5′-GCTACTACACCTAAATTCTAACC-3′	
Cytochrome oxidase 1 gene	JB3	5′-TTTTTTGGGCATCCTGAGGTTTAT-3′	[29]
	JB4.5	5′-TAAAGAAAGAACATAATGAAAATG-3′	
Cytochrome c oxidase subunit 1 gene	Cox1 I	5′-TTGTTATAAATTTTTGATTACTAAC-3′	[14]
	Cox1 II	5′-GACATAACATAATGAAAATG-3′	
Internal transcribed spacer 1 gene	NAP 9	5′-AACAGGTCTGTGATGCCCT-3′	[32]
	4S	5′-TCTAGATGCGTTCGAA(G/A)TGTCGATG-3′	
Gene for the diagnostic antigen Ts1	gTs14F	5′-ATGCGTGCCTACATTGTGCTTCTC-3′	[35]
	gTs14-R2	5′-GCAGTTTTTTTCTTAGGACCTTTGCAGTG-3′	

**Table 3:** LAMP assays for *Taenia solium* identification.

Methods	Marker	References
LAMP	mt COX1	[12, 13]
	Cathepsin L-like cysteine peptidase (clp)	[13]
Multiplex LAMP	mt COX1	[12]

**Table 4:** Application of RPA in diagnosis of helminthic and protozoan infections in humans and animals.

Parasite category	Parasite tested	Biological matrix	References
Protozoa	*Plasmodium falciparum*	Serum/whole blood	[22, 66]
	*Cryptosporidium parvum*	Stool	[55, 58]
	*Leishmania donovani*	Whole blood	[39, 67]
	*Toxoplasma gondii*	Soil	[59]
	*Trypanosoma cruzi*	Whole blood	[68]
	Giardi lambda	Stool	[58]
	*E.histolytica*	Stool	[69]

Helminths	*Schistosoma haematobium*	Urine	[20]
	*Schistosoma japonicum*	Stool	[60]
	*Schistosoma mansoni*	Stool	[18]
	*Fasciola hepatica*	Stool	[65]

**Table 5:** Comparison between established PCR and most common isothermal amplification methods.

	PCR	LAMP	RPA
Sample processing prior to amplification	Nucleic acid extraction required	Amplification from crude samples possible	Amplification from crude samples possible [22]
Simplicity of operation	Complex	Comparatively easier to setup than PCR	Relatively easier to setup than PCR
Cost	$6–7.7 [70]	$0.71–2	$4.3−4.7 [64, 69]
Stability of reagent	Cold chain required for enzymes	Cold chain required for enzymes	Reagents available as dry pellet [17]
		Reagents available as dry pellet [52, 53]	
Skills required	High	Moderate	Moderate
Reaction time	2 h [14]	60−90 mins [13, 21]	15−30 mins [22]
Amplification temperature	Varying [32, 33]	65°C [43]	25−42°C [17, 22]
Sensitivity	High	High	High
Specificity	High	High	High
Limit of detection	1 ng [25, 70]	5 eggs/g of feces [13]	Unknown
*Taenia solium* identification	Yes [14, 26, 27]	Yes [12, 13, 42]	Possible
Field tested	No	Yes	No
Product developer	Qiagen, Hilden, German [70]	New England Biolabs, Hitchin, UK	TwistDx, Cambridge, UK [20]
